# Comparison of culture, confocal microscopy and PCR in routine hospital use for microbial keratitis diagnosis

**DOI:** 10.1038/s41433-021-01812-7

**Published:** 2021-11-05

**Authors:** Jeremy J. Hoffman, John K. G. Dart, Surjo K. De, Nicole Carnt, Georgia Cleary, Scott Hau

**Affiliations:** 1 Moorfields Eye Hospital NHS Foundation Trust, London, UK; 2 International Centre for Eye Health, London School of Hygiene and Tropical Medicine, London, UK; 3 National Institute of Health Research (NIHR) Biomedical Research Centre at Moorfields Eye Hospital NHS Foundation Trust and UCL Institute of Ophthalmology, London, UK; 4 Department of Microbiology, University College London Hospitals NHS Foundation Trust, London, UK

**Keywords:** Corneal diseases, Diagnosis

## Abstract

**Aims:**

To evaluate the sensitivity and specificity of polymerase chain reaction (PCR), in vivo confocal microscopy (IVCM) and culture for microbial keratitis (MK) diagnosis.

**Methods:**

Retrospective review of PCR, IVCM and culture results for MK diagnosis at Moorfields Eye Hospital between August 2013 and December 2014.

**Results:**

PCR results were available for 259 MK patients with concurrent culture for 203/259 and IVCM for 149/259. Sensitivities and specificities with 95% confidence intervals [95% CI] were calculated for *Acanthamoeba* keratitis (AK) and fungal keratitis (FK), by comparison with culture, for both IVCM and PCR. For AK, FK and bacterial keratitis (BK) sensitivities were calculated, for each diagnostic method, by comparison with a composite reference standard (a positive result for one or more of culture, PCR or IVCM having a specificity of 100% by definition). For the latter, sensitivities with [95% CI] were: for AK, IVCM 77.1% [62.7–88.0%], PCR 63.3% [48.3–76.6%], culture 35.6 [21.9–51.2]; for FK, IVCM 81.8% [48.2–97.7%], PCR 30.8% [9.09–61.4%], culture 41.7% [15.2–72.3%]; for BK, PCR 25.0% [14.7–37.9%], culture 95.6% [87.6–99.1%].

**Conclusion:**

IVCM was the most sensitive technique for AK and FK diagnosis but culture remains our gold standard for BK. These findings reflect results to be expected from service providers to UK ophthalmology units and demonstrates the need at our centre for ongoing diagnostic result audit leading to the potential to improve PCR diagnosis. Both FK and AK are now common in the UK; ophthalmology units need to have all these techniques available to optimise their MK management.

## Introduction

Microbial keratitis (MK) can be caused by a diverse range of micro-organisms; accurate, early diagnosis of the causative organism is crucial to the choice of an appropriate antimicrobial treatment that is required for a good outcome. Empirical treatment for MK is widely used, without investigations, despite the overlap of clinical signs for the major groups of causative organisms; bacteria, *Acanthamoeba* and fungi, for which treatments are quite different.

The traditional ‘gold standard’ for diagnosing MK is microbiological diagnosis with microscopy and culture. However, the sensitivity of culture is poor with culture-positive rates ranging from 32.6 to 79.4% [1], increasing with the size of the ulcer [2]. Microscopy of stained corneal tissue (corneal biopsy and/or smear) achieves rates of 27.3 to 61.6% depending on the stain used and organism being identified [1]. Alternative tools, including polymerase chain reaction (PCR) and in vivo confocal microscopy (IVCM), have been developed to aid in the diagnosis of MK and are used in combination with culture and microscopy in some tertiary referral centres to help improve diagnostic precision in terms of sensitivity and specificity.

Although a number of studies have investigated the sensitivity and specificity of culture, PCR and IVCM for either fungal keratitis (FK) or *Acanthamoeba* keratitis (AK) [1, 3–8], these have often focused on one diagnostic modality for comparison, or have been specific to either bacterial keratitis (BK), FK or AK. The purpose of this study was to compare the sensitivity and specificity of IVCM, PCR, and corneal cultures, in routine clinical practice at our tertiary referral centre and using our external pathology service providers, for a consecutive cohort of patients presenting to our centre with MK of any cause. These patients include both primary and tertiary referrals.

## Subjects and methods

### Participants

This study was approved by Moorfields Eye Hospital (MEH) Clinical Audit and Assessment Committee (Ref: CA14/CED/38) and adhered to the tenets of the Declaration of Helsinki as a retrospective, observational case note review of patients who attended MEH with MK between 14th August 2013–6th December 2014. All three investigations (culture, PCR and IVCM) were being performed routinely at this point, and this study was performed to audit their results a few months after PCR was introduced. The inclusion criteria were patients with MK during this period who had undergone PCR.

### Culture and PCR

Patients with MK attending MEH are assessed using an in-house protocol available on the Microguide App (http://www.microguide.eu/services/mobile/), known as the Moorfields Emergency Guideline App. PCR, culture and IVCM are carried out on presentation if there is severe disease (a large ulcer or abscess with or without a hypopyon or ring infiltrate) or, for early disease, having features suggestive of non-bacterial keratitis or ‘atypical keratitis’. These investigations are also performed when keratitis cases are unresponsive to therapy.

PCR samples were collected by corneal swabs and sent to an external laboratory for analysis (Micropathology Ltd, Coventry, UK). The assays requested included 16S rRNA PCR (pan-bacterial), 18S rRNA PCR (pan-fungal), *Acanthamoeba* species-specific PCR, *Aspergillus* genus DNA PCR, *Candida albicans* DNA PCR, cytomegalovirus DNA PCR, Epstein-Barr virus DNA PCR, Group A *Streptococcus* DNA PCR, Group B *Streptococcus* DNA, Herpes Simplex Virus DNA PCR, *Mycobacterium* genus DNA PCR, varicella zoster virus DNA PCR and *Staphylococcus* DNA PCR. Corneal scrapings were collected in duplicate for microscopy and culture using fresh 21-gauge needles after anaesthetizing the eye with topical 0.5% proxymetacaine hydrochloride (Bausch & Lomb, U.K. Ltd). These were inoculated directly onto culture media (blood agar, Sabouraud’s dextrose agar for fungi, brain–heart-infusion broth and *Escherichia coli*-seeded non-nutrient agar subsequently seeded with *Escherichia coli* for *Acanthamoeba*) and sent for processing and reporting by an external service (The Doctors Laboratory Ltd, London, UK).

### In vivo confocal microscopy

IVCM was performed by trained experienced operators using the HRT II/RCM confocal microscope (Heidelberg Engineering, Dossenheim, Germany) using a previously described standard operating procedure [8]. Findings indicative of *Acanthamoeba* or FK were as previously reported and classified accordingly [4, 7, 9]. Bacteria, with the exception of *Nocardia spp*., are too small to be detected by IVCM [10]. All the images were reviewed and classified into the various type of keratitis, in a masked fashion, by one experienced observer (SH).

### Disease definition and analysis

Disease definition was based on a positive diagnosis using culture, PCR or IVCM. To ensure we were assessing the sensitivity and specificity of investigations, the clinical findings and patient response to antimicrobial treatment were not used in the analysis or for disease definition. The diagnostic capability of the various tests was evaluated by determining: (1) the sensitivity and specificity of PCR and IVCM in comparison to culture and (2) if a composite reference standard, in which a positive result from one or more of the techniques confirms the diagnosis, is superior to the historical ‘gold’ standard of culture. The reference standards for comparison are defined as follows:

- Culture: based on a positive result obtained from culture only.
- PCR: positive result from PCR only.
- IVCM: organism identified on imaging.
- Composite MK diagnosis: a positive result for at least 1 of culture, PCR or IVCM [8].

### Statistical analysis

For one analysis we have compared PCR, IVCM and culture for *Acanthamoeba a*nd filamentous fungus using each as a reference standard for comparison with the others. For the other analysis we have compared the results for Culture, PCR and IVCM (the latter not for bacteria) with the composite reference standard.

Data were collated in Microsoft Excel 2019 and analysed using STATA 15. We calculated sensitivity and specificity values, including exact binomial confidence intervals, for each diagnostic modality compared to the various reference standards.

## Results

A total of 259 patients with unilateral MK [52.9% female; mean age 52.7 years (SD 20.8; range 6–108)] had PCR samples analysed.

**Fig. 1:**
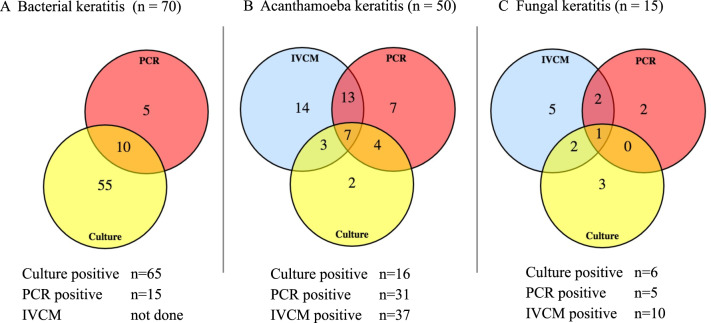
Venn diagram showing the number of cases that were positive for bacteria, *Acanthamoeba* and fungal keratitis using culture, polymerase chain reaction (PCR) and in vivo confocal microscopy (IVCM). Cases that were positive for more than one test are given within the overlapping areas. **A** Investigations that were positive for the 70 cases of bacterial keratitis including mixed infections. Note IVCM not included for bacterial keratitis; **B** Investigations that were positive for the 50 cases with Acanthamoeba keratitis including mixed infections; **C** Investigations that were positive for the 15 cases with fungal keratitis including mixed infections and the one case of *Candida dubliensis* infection, which was positive in all three investigations.

Table 1 describes the organisms detected, categorised by microbial group, for 259 patients with a clinical diagnosis of microbial keratitis using culture and/or PCR and/or IVCM. No organisms were detected in 128/259 (49.4%) of cases by any technique. PCR was used in all 259 cases, 203/259 had PCR and culture, and 149/259 had PCR and IVCM. However, although the numbers for culture and IVCM were fewer than the totals tested by PCR, most cases that were positive using a composite diagnosis (all techniques combined) had had all the tests performed—the few exceptions are mentioned in the text describing results for individual groups of organisms below. For all tests combined (any test positive) 131/259 subjects (50.6%) had positive results. The positive results by detection method for each category of organism are also shown in Table 1 and are summarised in Fig. 1, in which the Venn diagrams show the numbers positive by one or more tests.

**Table 1:** Organism(s) detected in 259 keratitis patients categorised by group of organism and method of detection for culture, polymerase chain reaction (PCR) and in vivo confocal microscopy (IVCM) both separately and for the composite diagnosis using all methods combined.

		Diagnostic methods							
		All methods combined Culture ± PCR ± IVCM (Composite microbial keratitis diagnosis)		Culture alone		PCR alone		IVCM alone	
Numbers of subjects tested		*n*	(%)	*n*	(%)	*n*	(%)	*n*	(%)
Total number		259	(100)	203	(100)	259	(100)	149	(100)
For Bacteria				203	(100)	189	(73.0)	n/a	
For Acanthamoeba				203	(100)	175	(67.6)	149	(100)
For Filamentary fungus				203	(100)	180	(69.4)	149	(100)
Total negative		128/259	(49.4)	116	(57.1)	195	(75.3)	102	(68.5)
Negative for Bacteria				135/203	(66.5)	129/195	(66.2)	n/a	
Negative for Acanthamoeba				158/203	(77.8)	126/195	(64.6)	101	(67.8)
Negative for Filamentary fungus				190/203	(93.6)	166/195	(85.1)	137	(91.9)
Total Positive (yield)		131/259	(50.6)	87/203	(42.9)	64	(24.7)	47	(31.5%)
**Positive results (yield) by organism groups**				**Bacteria, fungi and Acanthamoeba were identified by more than one method; the numbers identified by multiple methods are described in Fig.** 1					
				**Culture positive**		**PCR positive**		**IVCM positive**	
*Monomicrobial*	Bacteria	54	(20.8)	50/53	(94.3)	13/49	(26.5)	not applicable (n/a)	
Filamentary Fungus		10	(3.9)	4/9	(44.4)	2/9	(22.2)	7/9	(77.8)
Yeast		1	(0.4)	1/1	(100)	1/1	(100)	1/1	(100)
Acanthamoeba		38	(14.7)	15/33	(45.5)	24/37	(64.9)	26/36	(72.2)
Herpes simplex virus		10	(3.9)	n/a		10/10	(100)	n/a	
Varicella zoster virus		2	(0.8)	n/a		2/2	(100)	n/a	
*Polymicrobial*	Bacteria ***WITH***	4	(1.5)	3/3	(100)	1/2	(50)	n/a	
	Filamentary Fungal^a^			1/3	(33.3)	2/2	(100)	2/2	(100)
	Bacteria ***WITH***	12	(4.6)	12/12	(100)	1/9	(11.1)	n/a	
	Acanthamoeba			1/12	(8.3)	7/9	(77.8)	11/12	(91.7)

^a^All mixed bacterial/fungus infections were filamentary; there were no mixed bacterial/yeast infections in the course of this study.

### Bacterial keratitis

There were 54 monomicrobial bacterial keratitis cases, of which one did not undergo culture but in which PCR was positive (*Klebsiella spp*.). IVCM was not performed routinely for BK as the resolution is inadequate to visualise bacteria other than *Nocardia* sp. or to visualise infectious crystalline keratopathy (ICK). In our cohort, there were three cases of ICK imaged by IVCM; two were confirmed as bacterial on culture and one as *Acanthamoeba* by PCR. There were no cases of *Nocardia* in our cohort. Bacteria were also involved in 16 cases of polymicrobial keratitis (3 with filamentous fungus and 12 with *Acanthamoeba)* totalling 70 cases in which bacteria were a cause. Table 1 and Fig. 1A show that culture was positive in 65/70 (92.8%) of BK cases compared to PCR in 15/70 (21.4%). Figure 1A shows culture negative BK cases and the one case that did not undergo culture were diagnosed by PCR alone in only 5/70 (7.1%) cases. Supplementary Table 1 lists the bacterial organisms identified by culture and which of these were also detected by PCR.

### Acanthamoeba keratitis

Of the 50 cases of AK there were 38 monomicrobial and 12 polymicrobial in combination with BK. Culture was not performed for five cases, PCR not analysed for one case and IVCM not carried out for two cases. Table 1 and Fig. 1B show that IVCM was positive in 37/50 (74%) compared to PCR in 31/50 (62%) and culture in 16/50 (32%). Figure 1B shows that IVCM negative cases were diagnosed by PCR or culture, either alone or in combination, in 9/50 (18%) cases.

### Filamentous fungal keratitis and yeasts

Of the 14 cases of filamentous FK and 1 case of yeast keratitis, culture was not performed for one case of filamentous FK, PCR was not analysed for one and IVCM not carried out for one. The case of yeast FK *(Candida dubliniensis* by sequencing) was budding and identified by all three methods. Table 1 and Fig. 1C show that IVCM was positive in 10/15 (66.7%) identifying the highest proportion of cases compared to PCR in 5/15 (33.3%) and culture in 6/15 (40%). Figure 1C shows that IVCM negative cases were diagnosed with either PCR or culture in 5/15 (33.3%) of cases. Supplementary Table 2 lists the fungal organisms identified by culture and which of these were also detected by PCR and / or IVCM.

### Polymicrobial (mixed) infection

For mixed *Acanthamoeba* and bacterial infections, all 12 cases were culture positive for bacteria versus 1/9 tested by PCR. For polymicrobial fungal and bacterial infections, 3/3 were positive for bacteria on culture versus 1/3 for PCR. IVCM and PCR were more often positive than culture for the fungal and Acanthamoeba components of these polymicrobial cases. All the mixed bacterial/fungal infections were caused by filamentous fungi as opposed to yeasts.

### Viral keratitis

Of the 12 cases of viral keratitis, PCR detected herpes simplex virus in ten cases and varicella zoster virus (VZV) in two cases. This is the only diagnostic test available at our facility for viral keratitis, only performed if there is uncertainty regarding clinical diagnosis, and so was not used to generate sensitivity and specificity data.

### Sensitivity and specificity

Table 2 shows the summary statistics of the sensitivity and specificity values for PCR, IVCM, and cultures for both FK and AK, compared to the different reference standards. When compared to the ‘gold’ reference standard of culture, the sensitivity of PCR was higher for AK vs FK whereas IVCM was higher for FK vs AK, although the specificities were similar for both. The sensitivities of PCR and IVCM were similar for AK, whereas IVCM performed substantially better vs PCR for FK. The positive and negative test results for calculating these indices for this table are included in Supplementary Table 3.

**Table 2:** Sensitivity and specificity values with 95% confidence intervals (brackets) of culture, polymerase chain reaction and in vivo confocal microscopy compared to different reference standards for *Acanthamoeba* and filamentary fungus. Full details for calculating the sensitivity and specificity for each modality are given in online Supplementary Table 1.

Diagnostic method	Reference standard					
	Acanthamoeba			Filamentary fungus		
	Culture	PCR	IVCM	Culture	PCR	IVCM
**Culture**						
Sensitivity %	Referent	40.7 (22.4–61.2)	29.4 (15.1–47.5)	Referent	25.0 (0.63–80.6)	37.5 (8.52–75.5)
Specificity %	Referent	96.7 (91.8–99.1)	95.7 (89.5–98.8)	Referent	97.4 (93.4–99.3)	98.3 (94.1–99.8)
**PCR**						
Sensitivity %	73.3 (44.9–92.2)	Referent	69.0 (49.2–84.7)	20.0 (0.50–71.6)	Referent	30.0 (6.67–65.2)
Specificity %	88.1 (81.3–93.0)	Referent	83.5 (74.6–90.3)	98.0 (94.3–99.6)	Referent	100 (96.3–100)
**IVCM**						
Sensitivity %	71.4 (41.9–91.6)	69.0 (49.2–84.7)	Referent	60.0 (14.7–94.7)	100 (29.2–100)	Referent
Specificity %	78.9 (70.3–86.0)	83.5 (74.6–90.3)	Referent	95.9 (90.8–98.7)	93.4 (86.9–97.3)	Referent

*PCR* polymerase chain reaction, *IVCM* in vivo confocal microscopy.

Table 3 gives the sensitivity and specificity of PCR, IVCM and culture for AK and FK, as compared to the composite diagnosis reference standard. For AK, the highest sensitivity was with IVCM and lowest with culture. For FK, the highest sensitivity was also IVCM, however culture performed better than PCR. For bacteria, culture was substantially more sensitive than PCR. The specificities for all three diagnostic modalities were 100% when compared to the composite diagnosis reference standard because the composite reference by definition means that there will be no false positive results, as all positive results are included within the composite diagnosis category.

**Table 3:** Sensitivity and specificity values for detecting *Acanthamoeba*, filamentary fungus and bacteria using PCR, IVCM and culture compared to a composite diagnosis reference standard. The composite diagnosis reference standard is where an individual tests positive for an organism group (*Acanthamoeba*, bacteria or fungus) in one or more of the three diagnostic investigations. The sensitivity/specificity values are shown on the column to the right and the number of positive and negative test results, for the organism in question, are shown on the left.

Diagnostic Method^a^	Composite Diagnosis Reference Standard		Totals^b^	Indices	Value (% CI)
	Positive	Negative			
**Acanthamoeba (*n*** **= 50 detected including both mono and polymicrobial)**					
PCR					
Positive	31	0	31	Sensitivity %	63.3 (8.3–76.6)
Negative	18	126	144	Specificity %	100 (97.1–100)
Total	49	126	175		
IVCM					
Positive	37	0	37	Sensitivity %	77.1 (62.7–88.0)
Negative	11	101	112	Specificity %	100 (96.4–100)
Total	48	101	149		
Culture					
Positive	16	0	16	Sensitivity %	35.6 (21.9–51.2)
Negative	29	158	187	Specificity %	100 (97.7–100)
Total	45	158	203		
**Fungus (*n*** **= 14 detected including both mono and polymicrobial)**					
PCR					
Positive	4	0	4	Sensitivity %	30.8 (9.09–61.4)
Negative	9	167	176	Specificity %	100 (97.8–100)
Total	13	167	180		
IVCM					
Positive	9	0	9	Sensitivity %	81.8 (48.2–97.7)
Negative	2	138	140	Specificity %	100 (97.4–100)
Total	11	138	149		
Culture					
Positive	5	0	5	Sensitivity %	41.7 (15.2–72.3)
Negative	7	191	198	Specificity %	100 (98.1–100)
Total	12	191	203		
**Bacteria (*n*** **= 70 detected including both mono and polymicrobial)**					
PCR					
Positive	15	0	15	Sensitivity %	25.0 (14.7–37.9)
Negative	45	129	174	Specificity %	100 (97.2–100)
Total	60	129	189		
Culture					
Positive	65	0	65	Sensitivity %	95.6 (87.6–99.1)
Negative	3	135	138	Specificity %	100 (97.3–100)
Total	68	135	203		

*PCR* polymerase chain reaction, *IVCM* in vivo confocal microscopy; *CI* confidence interval.

^a^IVCM was not performed for cases of bacterial keratitis;

^b^The total number of individuals in the composite diagnosis reference standard differs for each organism group and investigation as not every individual had all three investigations performed. When comparing the tests for each organism group (bacteria, fungus or amoeba) to the composite reference standard, only the total number of patients who had the particular test in question being performed are included. Please refer to Table 1 for the number of diagnostic tests performed for each organism group in question.

## Discussion

This study compares the sensitivity and specificity of three diagnostic methods in use for the diagnosis of MK caused by bacteria, fungi and *Acanthamoeba* in the setting of both primary and tertiary ophthalmology referrals in London. IVCM is carried out ‘in-house’ and is not yet widely available in UK ophthalmology units but provided by a number of tertiary ophthalmic centres in major cities. We have shown that the overall diagnostic yield for a composite diagnosis (any method positive) was positive in 131/259 (50.6%) of patients with a clinical diagnosis of MK (Table 1). Of these there were 70 BK, 50 AK and 15 FK (Fig. 1). Although all diagnostic techniques contributed to the yield of positive diagnoses, culture provided the highest yields and sensitivity for BK, whereas IVCM provided the highest yields and sensitivity for both AK and FK, when compared to the composite diagnosis reference standard (Fig. 1 and Tables 1, 3). However, all appropriate techniques contributed additional diagnoses that would have not been made with only two of these three techniques. This study highlights the evidence for why having all three techniques available for the diagnosis on MK is required to optimise the yield of cases having a positive finding to aid diagnosis and management. Given the results of this study, clinicians who currently only have access to smear microscopy and culture should therefore be aware of the real-world limitations of culture in terms of sensitivity and specificity particularly for non-bacterial keratitis. If there are any features suggesting an atypical infection, referral to a centre where further investigations can be carried out should be made.

BK accounted for the highest proportion of cases of MK (70/131 cases, 53.4%), and FK for the lowest in this study, in keeping with other studies at temperate latitudes [11–15]. *Acanthamoeba* keratitis was identified in 50/131 cases (38.2%) which is higher than reported in previous studies [11–13]. This is likely to be due to three reasons: the fact that a clinical diagnosis was not used in the analysis which will have resulted in failure to identify probable BK in many small bacterial keratitis lesions which are more often culture negative [16], that Moorfields is a tertiary referral centre [17, 18], and that the data for this study was collected during an outbreak of AK in the South East of the UK [17].

Limitations of this study are several. To ensure we were evaluating the yield, sensitivity and specificity of these techniques, the clinical diagnosis (clinical findings and response to antimicrobial treatment) was not used as a comparator and it is not possible to be certain of the cause of keratitis in the 128/259 (49.4%) of patients in whom all diagnostic tests were negative. The low total yield (50.6%) identified in our study may itself bias the results as we can be unsure of the diagnostic accuracy of the patients who tested negative to all three tests but yet had a clinical diagnosis of MK. Microscopy results were not included as these were not accessible. Our masked observer is very experienced in analysing IVCM keratitis images making extrapolation of these results to units having less experienced IVCM operators uncertain. Not every case had every investigation performed, although most cases with a positive diagnosis by one test also had the other tests performed (Table 1). As a result, the numbers of patients within the composite diagnosis reference group to whom the different diagnostic investigations compared to in Table 3 differ between organism group and type of investigation, which may result in some bias. The number of FK cases in this series was low, resulting in wide confidence intervals. Finally, the processing and reporting of the cultures and material provided for PCR was carried out by external providers, using assays which may have had differing performance characteristics compared to those used in studies carried out in research laboratories using custom primers [19], or techniques [20]. On the other hand, dedicated research microbiology laboratories are rarely available for clinical use which relies on service providers. As a result, the findings from this study represent what most ophthalmic units in the UK and elsewhere might expect from their external providers of microbiology facilities.

The strengths of this study include the use of a single experienced masked assessor for interpreting the IVCM images, with all imaging performed following the same standard operating procedure; the large sample size of our cohort; the use of a composite diagnosis for technique comparison and the pragmatic evaluation of clinical diagnostic services.

The overall yield of positive cultures of 87/203 (42.9%) is consistent with findings from the UK, where a large series of MK including very small ulcers found that most cases of MK were caused by bacteria and found the culture positive rate was as low as 1 379/4 229 (33%) [21]. This is as opposed to the rates for cultures from large ulcers in India, where FK is more common than BK and culture positive rates are generally higher, ranging from 51.9% (56/108) for ulcers ≥1.0 mm [22], up to as high as 76% (182/239) for ulcers ≥3.00 mm.

Supplementary Table 4 is a summary of studies comparing PCR, IVCM and culture/smear yields, sensitivity and specificity in microbial keratitis. For PCR the diagnosis rates vary depending on the reference standard used, the PCR primers, the organisms, the severity of disease, whether or not the patients have been treated with antimicrobials before the samples are taken, and the unquantifiable factors of the quality of the diagnostic facilities and the sample. The principal difference in sensitivity, specificity and yield, by comparison with the studies summarised here is the relatively low sensitivities in our study for fungal and bacterial diagnosis using PCR. PCR for bacterial keratitis has been reported as more sensitive than culture in a similar study to ours [23], but from a population having a higher positive diagnostic rate and one that includes smear microscopy as part of the composite reference (likely reducing the calculated sensitivity of culture), whilst another has shown similar PCR positive rates to culture positive rates [24]. In contrast, our study found bacterial PCR sensitivity of 25% (CI 14.7–37.9%) versus culture of 95.6% (CI 87.6–99.1%). The higher rates of PCR sensitivity for bacterial keratitis reported elsewhere may have resulted from PCR false positives due to the detection of non-pathogenic bacteria as shown by Kim et al. [22], or better culture in combination with less effective PCR in our laboratories. Broad range 16S PCR has a limit of detection of between 10^3^ and 10^4^ colony forming units per millilitre [25], and our poor PCR sensitivity may result from samples being sent from less keratitis with less severe disease at presentation and/or that the samples we send have less than the optimal amount of material available for PCR. PCR for fungal keratitis has also been shown by most studies to be more often positive and more sensitive than culture [20, 23, 26, 27], with sensitivities between 70% and 93% compared to that of culture of 43% to 57%; one study showed approximately the same yield using both techniques [28]. These findings compare to ours with a sensitivity for fungal PCR of 30.8% (CI 9.1–61.4%) versus culture of 41.7% (CI 15.2–17.3%); the low sensitivity of PCR again being potentially due to early disease at presentation or poor sampling, as well as a poor choice of primers. Analysis of PCR for *Acanthamoeba* keratitis compared to culture has given variable results in different studies. Two studies show PCR, depending on the PCR primer used, to be more sensitive than culture with sensitivities ranging from 65% to 95% versus culture at 73.7% [29], or with a higher yield of 53% to 73% versus culture of 55% whereas two others with high rates of culture or smears positive (70-80%) show a similar yield from PCR [30, 31]. Our study shows comparable findings with sensitivity of 63% for *Acanthamoeba* PCR compared to 35.6% for culture.

IVCM diagnostic sensitivity depends, as for PCR, on the reference standard used which has been culture and/or microscopy in three studies [3, 5, 7], these, with the addition of some clinically diagnosed cases in a fourth [32], and *Acanthamoeba* PCR in a fifth [33]. Two studies showed sensitivities of 80–90% with specificities of 80–90% for both AK and FK diagnosis [5, 7], similar to those for AK diagnosis in a third study [32]. However, a further study comparing IVCM diagnosis for *Acanthamoeba*, filamentous fungus, *Nocardia* and *Microsporidia* between five different observers found very variable results depending on the experience of the observer; the most experienced having a sensitivity of 55.8% and specificity of 84.2% [3]. Our findings in this study, using an experienced observer, are similar to these published studies having sensitivities [specificities] for *Acanthamoeba* and filamentous fungus respectively of 77.1% [100%] and 81.8% [100%]. The IVCM criteria used for AK diagnosis may also affect the sensitivity and specificity varying with the criteria used for diagnosis; those giving the highest sensitivity of 73.9% had the lowest specificity of 48.2% and those with the highest specificity of 98.2% having the lowest sensitivity of 15.2% [33]. However, sensitivity and specificity are not always of equal clinical importance; sensitivity may be deemed to be more useful than specificity in this case in order not to miss atypical organisms. This should be considered when interpreting the results of our study.

Our study shows what may be achieved for the diagnosis of MK using general (non-ophthalmic) culture and PCR service providers that are available to UK hospitals.

These findings may be useful to others as a benchmark for what can be expected for MK diagnosis in the UK and can be used to help improve the sensitivity of diagnostic tests for MK. Lastly, this study demonstrates that all three techniques are needed to optimise MK diagnosis and, given the increasing incidence of fungal and *Acanthamoeba* keratitis in the UK [21, 34], shows that these facilities should be made available to all UK ophthalmic units.

### Summary table

#### What was known before

- The traditional “gold standard” for diagnosing MK is microbiological diagnosis with microscopy and culture.
- In vivo confocal microscopy and Polymerase Chain Reaction (PCR) are potentially useful adjunctive tools to diagnosis.
- The purpose of this study was to compare the sensitivity and specificity of IVCM, PCR, and corneal cultures, in routine clinical practice.

#### What this study adds

- In vivo confocal microscopy was the most accurate tool for diagnosing *Acanthamoeba* and fungal keratitis compared to PCR and culture.
- PCR was more sensitive for *Acanthamoeba* than fungus.
- Culture performed best for bacterial keratitis.
- Ophthalmology units in the UK need to have all these techniques available to optimise their MK management.

## Supplementary information

Supplementary Table 1

Supplementary Table 2

Supplementary Table 3

Supplementary Table 4
