# Supplementary material for: Comparison of culture, confocal microscopy and PCR in routine hospital use for microbial keratitis diagnosis
Source: Eye (Lond). 2021 Nov 5;36(11):2172–8. doi: 10.1038/s41433-021-01812-7 (PMC9581916; doi:10.1038/s41433-021-01812-7)
Supplement: Supplementary file 1 — Supplementary Table 1 [file 41433_2021_1812_MOESM1_ESM.pdf]

Supplementary Table 1: Organisms detected for the 65 bacterial organisms identified by culture and whether these were positive by PCR. There were an additional four cases that were culture negative (*Abiotrophia defectiva, Streptococcus mitis, Staphylococcus spp.* & *Bradyrhizobium spp.)* and one case that was positive for PCR and not cultured (*Klebsiella spp.*)

**Organism**	**Number of cases**	**16S rRNA positive**	**Organism-specific PCR positive**	**Comment**
Stenotrophomonas maltophilia	1	0	0	
Staphylococcus aureus	6	0	0	1 PCR for HSV DNA
Staphylococcus epidermidis	11	0	1	3 mixed infections with *Acanthamoeba* DNA detected by PCR
Streptococcus pneumoniae	8	1	2	
Corynebacterium amycolatum	1	0	0	Fungal PCR positive (possible contaminant)
Achromobacter xylosoxidans	1	0	0	
Pseudomonas aeruginosa	9	3	0	
Staphylococcus wameri	2		0	1 mixed infection with *Acanthamoeba cysts on culture*
Moraxella nonliqueficians	1	0	0	
Moraxella catarrhalis	1	0	0	
Staph. capitis	7	0	0	1 PCR for HSV DNA
Mycobacterium abscessus	1	0	0	
Acinetobacter lwoffii	1	0	0	
Acinetobacter parvus	2	0	0	
Strep. oralis	2	2	0	
Pseudomonas spp.	1	0	0	
Corynebacterium afermentans	1	0	0	
Beta haemolytic *Streptococcus spp.*	1	0	0	
Enterococcus spp.	1	0	0	
Corynebacterium propinquum	1	0	0	
Pseudomonas oryszihabitans	1	0	0	
Corynebacterium spp.	1	0	0	
Entero cloacae	1	0	0	1 mixed infection with *Acanthamoeba* DNA detected by PCR
Coagulase negative *Staph. spp.*	1	0	0	1 mixed infection with *Acanthamoeba* DNA detected by PCR
Staphylococcus haemolyticus	1	0	0	1 mixed infection with *Acanthamoeba* DNA detected by PCR
Moraxella lacunata	1	1	0	

rRNA: ribosomal RNA; PCR: polymerase chain reaction; HSV: herpes simplex virus
