# Supplementary material for: Comparison of culture, confocal microscopy and PCR in routine hospital use for microbial keratitis diagnosis
Source: Eye (Lond). 2021 Nov 5;36(11):2172–8. doi: 10.1038/s41433-021-01812-7 (PMC9581916; doi:10.1038/s41433-021-01812-7)
Supplement: Supplementary file 2 — Supplementary Table 2 [file 41433_2021_1812_MOESM2_ESM.pdf]

Supplementary Table 2: Organisms detected for the 6 fungal organisms identified by culture and whether these were positive by PCR. There were an additional 4 cases that were culture negative, 2/4 positive for PCR alone (both *Cladosporium spp.*) and 2/4 positive for PCR and IVCM (*Aspergillus fumigatus* and *Fusarium spp.*)

**Organism**	**Number of cases**	**18S rRNA positive**	**Organism-specific PCR positive**	**IVCM Positive**
Candida dubliniensis	1	1	1	1
Scedosporium spp.	1	0	0	1
Unidentified filamentous fungus	2	0	0	1
Fusarium spp.	1	0	0	0
Acremonium spp.	1	0	0	0

rRNA: ribosomal RNA; PCR: polymerase chain reaction; IVCM: *in vivo* confocal microscopy
