# Supplementary material for: Comparison of culture, confocal microscopy and PCR in routine hospital use for microbial keratitis diagnosis
Source: Eye (Lond). 2021 Nov 5;36(11):2172–8. doi: 10.1038/s41433-021-01812-7 (PMC9581916; doi:10.1038/s41433-021-01812-7)
Supplement: Supplementary file 3 — Supplementary Table 3 [file 41433_2021_1812_MOESM3_ESM.pdf]

**Supplementary Table 3:** Sensitivity and specificity values with 95% confidence intervals (brackets) of culture, polymerase chain reaction and *in vivo* confocal microscopy compared to different reference standards for *Acanthamoeba* and filamentary fungus, expanding on the data presented in Table 2. The sensitivity/specificity values are shown on the column to the right and the number of positive and negative test results, for the organism in question, are shown on the left.

| **Diagnostic Modality** | **Culture Reference Standard** | | **Totals** | **Indices** | **Value (% CI)** |
|---|---|---|---|---|---|
| | Positive | Negative | | | |
| **Acanthamoeba (n=50 detected including both mono and polymicrobial)** | | | | | |
| **PCR** | | | | | |
| Positive | 11 | 16 | 27 | **Sensitivity %** | 73.3 (44.9-92.2) |
| Negative | 4 | 118 | 144 | **Specificity %** | 88.1 (81.3-93.0) |
| **Total** | 15 | 134 | 149 | | |
| **IVCM** | | | | | |
| Positive | 10 | 24 | 34 | **Sensitivity %** | 71.4 (41.9-91.6) |
| Negative | 4 | 90 | 94 | **Specificity %** | 78.9 (70.3-86.0) |
| **Total** | 14 | 114 | 128 | | |
| **Fungus (n=14 detected including both mono and polymicrobial)** | | | | | |
| **PCR** | | | | | |
| Positive | 1 | 3 | 4 | **Sensitivity %** | 20.0 (0.50-71.6) |
| Negative | 4 | 148 | 152 | **Specificity %** | 98.0 (94.3-99.6) |
| **Total** | 5 | 151 | 156 | | |
| **IVCM** | | | | | |
| Positive | 3 | 5 | 8 | **Sensitivity %** | 60.0 (14.7-94.7) |
| Negative | 2 | 118 | 120 | **Specificity %** | 95.9 (90.8-98.7) |
| **Total** | 5 | 123 | 128 | | |
| **Diagnostic Modality** | **PCR Reference Standard** | | **Totals** | **Indices** | **Value (% CI)** |
| | Positive | Negative | | | |
| **Acanthamoeba (n=50 detected including both mono and polymicrobial)** | | | | | |
| **Microbiology** | | | | | |
| Positive | 11 | 4 | 15 | **Sensitivity %** | 40.7 (22.4-61.2) |
| Negative | 16 | 118 | 134 | **Specificity %** | 96.7 (91.8-99.1) |
| **Total** | 27 | 122 | 149 | | |
| **IVCM** | | | | | |
| Positive | 20 | 16 | 36 | **Sensitivity %** | 69.0 (49.2-84.7) |
| Negative | 9 | 81 | 90 | **Specificity %** | 83.5 (74.6-90.3) |
| **Total** | 29 | 97 | 126 | | |
| **Fungus (n=14 detected including both mono and polymicrobial)** | | | | | |
| **Microbiology** | | | | | |
| Positive | 1 | 4 | 5 | **Sensitivity %** | 25.0 (0.63-80.6) |
| Negative | 3 | 148 | 151 | **Specificity %** | 97.4 (93.4-99.3) |
| **Total** | 4 | 152 | 156 | | |
| **IVCM** | | | | | |
| Positive | 3 | 7 | 10 | **Sensitivity %** | 100 (29.2-100) |
| Negative | 0 | 99 | 99 | **Specificity %** | 93.4 (86.9-97.3) |
| **Total** | 3 | 106 | 109 | | |

| Diagnostic Modality | IVCM Reference Standard | | Totals | Indices | Value (% CI) |
|---|---|---|---|---|---|
| | Positive | Negative | | | |
| **Acanthamoeba (n=50 detected including both mono and polymicrobial)** | | | | | |
| **Microbiology** | | | | | |
| Positive | 10 | 4 | 14 | **Sensitivity %** | 29.4 (15.1-47.5) |
| Negative | 24 | 90 | 114 | **Specificity %** | 95.7 (89.5-98.8) |
| **Total** | 34 | 94 | 128 | | |
| **PCR** | | | | | |
| Positive | 20 | 16 | 36 | **Sensitivity %** | 69.0 (49.2-84.7) |
| Negative | 9 | 81 | 90 | **Specificity %** | 83.5 (74.6-90.3) |
| **Total** | 29 | 97 | 126 | | |
| **Fungus (n=14 detected including both mono and polymicrobial)** | | | | | |
| **Microbiology** | | | | | |
| Positive | 3 | 2 | 5 | **Sensitivity %** | 37.5 (8.52-75.5) |
| Negative | 5 | 118 | 123 | **Specificity %** | 98.3 (94.1-99.8) |
| **Total** | 8 | 120 | 128 | | |
| **PCR** | | | | | |
| Positive | 3 | 0 | 3 | **Sensitivity %** | 30.0 (6.67-65.2) |
| Negative | 7 | 99 | 106 | **Specificity %** | 100 (96.3-100) |
| **Total** | 10 | 99 | 109 | | |

PCR = Polymerase chain reaction; IVCM = In vivo confocal microscopy; CI = Confidence interval.
*IVCM was not performed for cases of bacterial keratitis;
†Composite diagnosis reference standard is defined as a positive result for at least 1 of the following: culture, PCR or IVCM.
