# Supplementary material for: Comparison of culture, confocal microscopy and PCR in routine hospital use for microbial keratitis diagnosis
Source: Eye (Lond). 2021 Nov 5;36(11):2172–8. doi: 10.1038/s41433-021-01812-7 (PMC9581916; doi:10.1038/s41433-021-01812-7)
Supplement: Supplementary file 4 — Supplementary Table 4 [file 41433_2021_1812_MOESM4_ESM.pdf]

Supplementary Table 4

Author	Disease	Reference standard	Number of cases	PCR		IVCM		Culture		Smear		Comment
				Sensitivity % [specificity %]	Number positive/total (%)	Sensitivity % [specificity %]	Number positive/total (%)	Sensitivity % [specificity %]	Number positive/total (%)	Sensitivity % [specificity %]	Number positive/total (%)	
**PCR studies**												
Panda 2014	**BK**	Culture	122	na	56/122 (45.9%)	nd	nd	nd	53/122 (43.4%)	na	29/122 (23.8%)	Sensitivity and specificity are compared for PCR versus smears in this study
Tananuvat N 2012	**FK**	Clinical diagnosis confirmed	30	93 [100]	28/30 (93.3%)	nd	nd	43 [100]	12/30 (40%)	20 [100]	6/30 (20%)	PCR primers in detail; probably research not commercial
Kuo MT 2012	**FK**	Culture **and/or** "dot-assay" PCR with clinically consistent findings	20 of 50 fungus cases analysed	100 [96]	20/20 (100%)	nd	nd	56 [100]	10/20 (50%)	nd	nd	PCR was a “dot-assay” special technique. 50 cases evaluated in total of which 20 met FK criteria
Eleinen KG 2012	**FK**	Any test positive of culture or PCR from a series of 88 MK screened for FK and BK.	88 of which up to 40 were positive for Fungus	90.91%	40 (45.5%)	nd	nd	59.09%	26 (29.5%)	65.91%	29/88 (33%)	Used pan-fungal and pan-bacterial PCR primers
	**BK**		88 of which up to 29 were positive for bacteria	87.88%	29/88 (33%)	na	na	57.58%	19/88 (21.6%)	33.30%	11/88 (12.5%)	
Ferrer C 2011	**FK**	Any test positive with confirmation by clinical outcome	20	nd	18/20 (90%)	nd		nd	16/20 (80%)	nd		
Vengayil 2008	**FK**	Clinical diagnosis	40	70% [56.7%]	20/40 (50%)	nd	nd			60% [66.7%] for KOH stain; 40% {66.7%] for Gram's stain	16/40 (40%) for KOH; 14/40 (35%) for Gram's	
Boggild A 2009	**AK**	Any 2 tests of microscopy, culture or PCR positive	107 suspected cases of which 20 were confirmed	Nelson primers90% (95% CI, 76.9 to 100%) [90.8%] with Nelson primers **AND** with JDP primers 65% (95% CI, 44.1 to 85.9%) [100%]	Nelson Primers 26/81 (32%) AND with JDP primers 13/94 (13.8%)	nd	nd	73.7% [100%]	14/91 (15.4%)	55% (100%)	11/96 (11.5%)	
Kowalski 2015	**AK**	PCR and/or culture positive	21	nd	18/21 (85.7%)	nd	nd	nd	17/21 (80.9%)	nd	nd	Using a validated PCR with sensitivity 100%, specificity 97%, positive predictive value of 75% and negative predictive value of 100%. 4/21 were PCR positive, culture negative and 3/21 culture positive, PCR negative
Lau 2015	**AK**	Qvarnstrom PCR primer	49	Riviere 78.6% [91.4%]; Nelson 64.3% [88.6%, JDP 92.9% [91.4%]	Riviere 14/49; Nelson 13/49; JDP 16/49	nd	nd	nd	nd	nd	nd	49 strains of *Acanthamoeba* (40 clinical and 9 reference using 4 different PCR primers (Qvarnstrom used by the CDC, Riviere, Nelson and JDP). Positive and negative predictive values were very similar for all three
Yera 2006	**AK**	Culture and/or smear and/or clinical	12 of 513 cases referred	94% with one or more of Nelson, ACARNA and JDP primers	10/12 Nelson, 10/12 ACARNA, 10/12 JDP	nd	nd	7%	1/12	33%	04-Dec	Although the yield for the three different PCR primers was the same the positives were not all for the same samples; they correlated in 8/12 and at least one was positive for all 12 cases although there was one false positive for a Nelson primer
Pasricha 2003	**AK**	Any test positive of culture , smear or PCR	10	87.5% [97.8%]		nd	nd	nd	8/10 (80%)	87.5% [95.6%]	7/10 (70%)	Positive predictive value of PCR 87.5%, negative predictive value 97.8%. No cases diagnosed by PCR not diagnosed by smears or culture
Lehmann 1998	**AK**	Clinical diagnosis	20	nd	16/19 (84%) with 2 primers. Primer P1 12/19 (53%) and P2 14/19 (73%)	nd	nd	nd	11/20 (55%)	nd	na	10/10 culture positive tested by PCR were all positive by PCR and 3/14 (21%) positive on histopathology
**IVCM studies**												
Chidambaram J 2016	**FK**	Culture and/or histology	176/239 (74%)	nd	na	85.7% [81.4%]		na	na	na	na	Inclusion criteria was for large ulcers only ≥3mm probably accounting for the high rate of culture and or histology positive cases; culture and/or histology negative rate very low at 27/239 (11%). Results for IVCM are pooled sensitivity and specificity for 5 graders. An additional 19/239 cases were positive for bacterial keratitis and not analysed by IVCM
	**AK**		17/239 (7%)	nd	na	88.2% [98.2%]		na		na		
Vaddavalli P 2011	**FK**	Culture and/or histology of 148 cases of which 93 were positive for FK and 93 was used as the reference	93	nd	na	89.2% [92.7%]	83/93 (89.2%)	na	na	na	na	148 cases of which 93 FK and 10 AK. Some were BK but number not stated; these used as controls. Only 45/148 were negative for AK or FK on culture/histology (very high)
	**AK**		10/148	nd	na	80% [100%]	8/10 (80%)	na		na		
Tu 2008	**AK**	Culture, smear and a combined standard of these with clinical	53	nd	nd	90.6% (CI 79.3%-96.9%) [100% (95% CI 95%-100%)] for combined ref standard	48/53 (88.6%)	52.8% (95% CI 38.6%-66.7%)	23/42 (54.8%)	na	30/41 (73.1%)	
Hau 2010	**FK, AK, Ms, Noc**	Culture and/or histology with clinical confirmation: 62/105 (59%) cases were positive by these criteria. Only the 63 positive cases were used of which 19 were BK and used as controls	11 fungal; 1 mixed FK and BK; 27/62 AK; 3/62 microsporidia; 2 nocardia and 19 BK	nd		55.8% [84.2%] highest values for IVCM	na	na		na		Comparison of confocal for the diagnosis of several different organisms causing keratitis (including *Microsporidia* and *Nocardia*). Results varied widely between 5 different observers
De Craene 2017	**AK**	50 patients with PCR positive AK and 50 controls with a variety of causes of non-AK keratitis	50	na	na	73.9% [48.2%] for Category 1 features and 15.2% [98.2%] for Category 2 features (see Comment for these)	na	na	na	na	na	Identifies features on IVCM that are associated with PCR-positive AK including target images, trophozoite-like images and clusters of of highly reflective objects. However finds the overall sensitivity low with positive predictive value 87.5% and negative predictive value 58.5%. Category 1 features: round or ovoid hyporeflective images with no double wall, target images, clusters, or trophozoites. Category 2 features: target images, clusters or trophozoites

Legend

Results for bacterial keratitis studies are highlighted in blue, *Acanthamoeba* keratitis in pink, filamentary fungal keratitis in yellow and combined studies in grey. BK, bacterial keratitis; AK, *Acanthamoeba* keratitis; FK, filamentary fungal keratitis; Ms, *Microsporidia* keratitis; Noc, *Nocardia* keratitis; PCR, polymerase chain reaction; IVCM, *in vivo* confocal microscopy; nd, not done; na, not applicable; KOH, potassium hydroxide smear microscopy staining

References

Boggild, A. K., D. S. Martin, T. Y. Lee, B. Yu and D. E. Low (2009). "Laboratory diagnosis of amoebic keratitis: comparison of four diagnostic methods for different types of clinical specimens." J Clin Microbiol **47**(5): 1314-1318.
Chidambaram, J. D., N. V. Prajna, N. L. Larke, S. Palepu, S. Lanjewar, M. Shah, S. Elakkiya, P. Lalitha, N. Carnt, M. H. Vesaluoma, M. Mason, S. Hau and M. J. Burton (2016). "Prospective Study of the Diagnostic Accuracy of the In Vivo Laser Scanning Confocal Microscope for Severe Microbial Keratitis." Ophthalmology **123**(11): 2285-2293.
De Craene, S., J. Knoeri, C. Georgeon, P. Kestelyn and V. M. Borderie (2018). "Assessment of Confocal Microscopy for the Diagnosis of Polymerase Chain Reaction-Positive Acanthamoeba Keratitis: A Case-Control Study." Ophthalmology **125**(2): 161-168.
Eleinen, K. G., A. A. Mohalhal, H. E. Elmekawy, A. M. Abdulbaki, A. M. Sherif, R. H. El-Sherif and E. M. Abdul Rahman (2012). "Polymerase chain reaction-guided diagnosis of infective keratitis - a hospital-based study." Curr Eye Res **37**(11): 1005-1011.
Ferrer, C. and J. L. Alio (2011). "Evaluation of molecular diagnosis in fungal keratitis. Ten years of experience." J Ophthalmic Inflamm Infect **1**(1): 15-22.
Hau, S. C., J. K. Dart, M. Vesaluoma, D. N. Parmar, I. Claerhout, K. Bibi and D. F. Larkin (2010). "Diagnostic accuracy of microbial keratitis with in vivo scanning laser confocal microscopy." Br J Ophthalmol **94**(8): 982-987.
Kowalski, R. P., M. A. Melan, L. M. Karenchak and A. Mammen (2015). "Comparison of Validated Polymerase Chain Reaction and Culture Isolation for the Routine Detection of Acanthamoeba From Ocular Samples." Eye Contact Lens **41**(6): 341-343.
Kuo, M. T., H. C. Chang, C. K. Cheng, C. C. Chien, P. C. Fang and T. C. Chang (2012). "A highly sensitive method for molecular diagnosis of fungal keratitis: a dot hybridization assay." Ophthalmology **119**(12): 2434-2442.
Lehmann, O. J., S. M. Green, N. Morlet, S. Kilvington, M. F. Keys, M. M. Matheson, J. K. Dart, J. I. McGill and P. J. Watt (1998). "Polymerase chain reaction analysis of corneal epithelial and tear samples in the diagnosis of Acanthamoeba keratitis." Invest Ophthalmol Vis Sci **39**(7): 1261-1265.
Pasricha, G., S. Sharma, P. Garg and R. K. Aggarwal (2003). "Use of 18S rRNA gene-based PCR assay for diagnosis of acanthamoeba keratitis in non-contact lens wearers in India." J Clin Microbiol **41**(7): 3206-3211.
Tananuvat, N., K. Salakthuantee, N. Vanittanakom, M. Pongpom and S. Ausayakhun (2012). "Prospective comparison between conventional microbial work-up vs PCR in the diagnosis of fungal keratitis." Eye (Lond) **26**(10): 1337-1343.
Tu, E. Y., C. E. Joslin, J. Sugar, G. C. Booton, M. E. Shoff and P. A. Fuerst (2008). "The relative value of confocal microscopy and superficial corneal scrapings in the diagnosis of Acanthamoeba keratitis 1." Cornea **27**(7): 764-772.
Vaddavalli, P. K., P. Garg, S. Sharma, V. S. Sangwan, G. N. Rao and R. Thomas (2011). "Role of confocal microscopy in the diagnosis of fungal and acanthamoeba keratitis." Ophthalmology **118**(1): 29-35.
Vengayil, S., A. Panda, G. Satpathy, N. Nayak, S. Ghose, D. Patanaik and S. Khokhar (2009). "Polymerase chain reaction-guided diagnosis of mycotic keratitis: a prospective evaluation of its efficacy and limitations." Invest Ophthalmol Vis Sci **50**(1): 152-156.
